# Apoptosis contributes to protect germ cells from the oogenic germline starvation response but is not essential for the gonad shrinking or recovery observed during adult reproductive diapause in *C*. *elegans*

**DOI:** 10.1371/journal.pone.0218265

**Published:** 2019-06-13

**Authors:** E. Carranza-García, R. E. Navarro

**Affiliations:** Departamento de Biología Celular y Desarrollo, Instituto de Fisiología Celular, Universidad Nacional Autónoma de México, Ciudad de México, México; East Carolina University, UNITED STATES

## Abstract

When *C*. *elegans* hermaphrodites are deprived of food during the mid-L4 larval stage and throughout adulthood, they enter an alternative stage termed “adult reproductive diapause (ARD)” in which they halt reproduction and extend their lifespan. During ARD, germ cell proliferation stops; oogenesis is slowed; and the gonad shrinks progressively, which has been described as the “oogenic germline starvation response”. Upon refeeding, the shrunken gonad is regenerated, and animals recover fertility and live out their remaining lifespan. Little is known about the effects of ARD on oocyte quality after ARD. Thus, the aim of this study was to determine how oocyte quality is affected after ARD by measuring brood size and embryonic lethality as a reflection of defective oocyte production. We found that ARD affects reproductive capacity. The oogenic germline starvation response protects oogenic germ cells by slowing oogenesis to prevent prolonged arrest in diakinesis. In contrast to a previous report, we found that germ cell apoptosis is not the cause of gonad shrinkage; instead, we propose that ovulation contributes to gonad shrinkage during the oogenic germline starvation response. We show that germ cell apoptosis increases and continues during ARD via *lin-35/Rb* and an unknown mechanism. Although apoptosis contributes to maintain germ cell quality during ARD, we demonstrated that apoptosis is not essential to preserve animal fertility. Finally, we show that IIS signaling inactivation partially participates in the oogenic germline starvation response.

## Introduction

**Fig 1:**
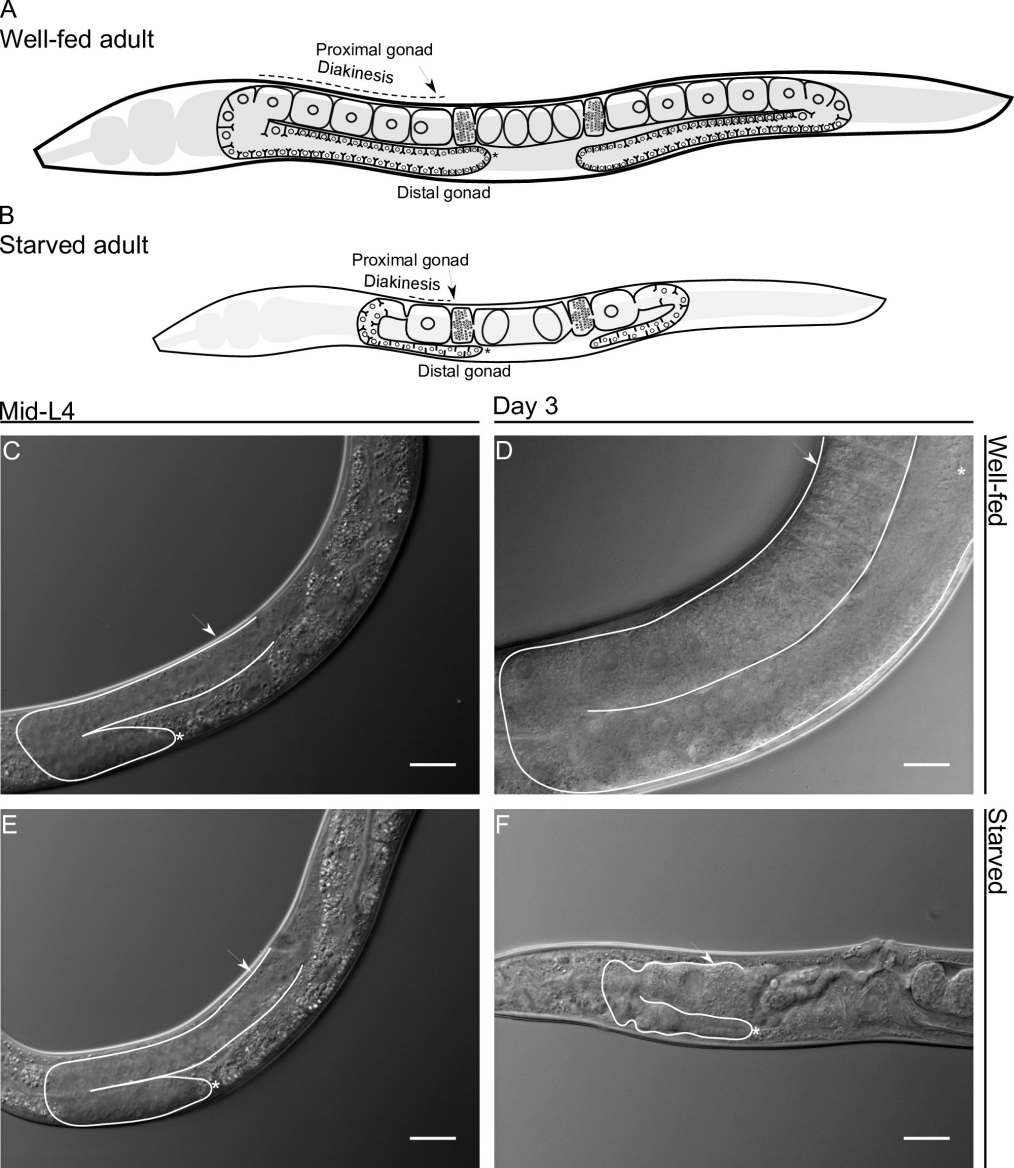
Comparison between well-fed and starved adult hermaphrodite gonad arms in *Caenorhabditis elegans*. Schematic representation of well-fed (A) and starved (B) adult hermaphrodites. (C, E) Nomarski image of mid-L4 gonad arms. (D) Nomarski image of a hermaphrodite that was well-fed for 3 days. (F) Nomarski image of a hermaphrodite that was starved for 3 days from mid-L4. In all images one gonad arm is outlined in white; the distal gonad is marked with an asterisk (*); and the arrow points to the proximal gonad. Scale bar = 20 μm.

To ensure species continuity, animals have developed mechanisms for protecting germ cells during stressful conditions. The *C*. *elegans* hermaphrodite germline serves as an excellent model for studying cell biology. In *C*. *elegans* hermaphrodites, 2 identical U-shaped gonad arms contain germ cells (Fig 1A). Under control conditions, L4 hermaphrodites (Fig 1C and 1E) produce approximately 40 germ cells that give rise 160 spermatids per gonad arm, which are stored within each spermatheca. Thereafter, during the adult stage, the remaining germ cells either differentiate into oocytes or are eliminated by physiological germline apoptosis [1, 2]. Physiological apoptosis is an essential mechanism for maintaining oocyte quality during oogenesis, as it promotes the allocation of nutrients to growing oocytes [3]. The most proximal oocytes arrest in diakinesis until they are fertilized, then complete meiosis and begin embryogenesis [3, 4] (Fig 1A and 1D). During its fertile period, a hermaphrodite produces approximately 300 new organisms in 3 days by self-fertilization with very low embryonic lethality (approx. 1–2 dead embryos/animal). Then, they cease laying eggs and live for 15 more days [4].

The short, highly stereotyped reproductive cycle of *Caenorhabditis elegans* can be altered when animals are deprived of food and enter into reversible states of growth arrest or diapause, depending on the stage in which they are deprived of food [5]. Animals subjected to high temperatures, crowding or fasting during the L1-L2 stage transition develop into a well-studied alternative larval stage known as the “dauer” stage. During dauer diapause, animals seal their orifices and form a thick impermeable cuticle, allowing them to endure stress for months [6].

When mid-L4 larvae or adult hermaphrodites face starvation conditions, they enter into adult reproductive diapause (ARD), characterized by delayed reproduction and an extended lifespan [7, 8]. ARD is a not yet fully elucidated form of diapause and differs from dauer diapause since animals starved at a low population density can enter and maintain this alternate developmental stage [8]. It has been observed that when starvation begins during the late-L4 or adult stage, embryos are retained *in utero*, hatch and cause the hermaphrodite’s death [7]; however, this fate can be avoided if embryo viability is inhibited [8]. During ARD, the hermaphrodites’ gonad shows a remarkable change; it shrinks in size progressively and the number of germ cells decreases to a small pool of ~35 cells [7] (Fig 1B and 1F). Starvation causes subsequent halting of the cell cycle in mitotically proliferating germ cells, which remain quiescent until conditions are restored [9]. The starved hermaphrodites form a single oocyte per gonad arm and usually carry 1–2 developing embryos within the uterus. These embryos are the result of delayed ovulation and fertilization, which occur approximately every 8 h ([8] and Fig 1B and 1F).

Remarkably, gonad shrinking and delayed reproduction are reversible [7, 8]. Following refeeding, germ cells resume their cell-cycle progression and start dividing, causing gonad regeneration, which, at least morphologically under the microscope, resembles a young adult gonad that never has been starved [7–9]. Hermaphrodites recover fertility and produce progeny by self-fertilization or mating [7]. Angelo and van Gilst (2009) proposed that apoptosis plays a crucial role during ARD, since apoptosis-defective mutants subjected to starvation from the mid-L4 stage do not reduce their gonad size and are unable to recover fertility after ARD [7].

ARD extends not only the total lifespan but also the reproductive period of animals [7]. In *C*. *elegans*, as in many other organisms, fertility declines with age [10], and oocyte quality is greatly affected during aging [3]. Adult reproductive diapause has been proposed as an anti-aging mechanism for protecting the germline [7]. Despite the numerous unexplained aspects of this phenomenon, whether ARD protects oocyte quality and the mechanisms that control this phenomenon have not yet been determined.

In this report, we use brood size and embryonic lethality to reflect defective oocyte production to investigate the effects of ARD on oocyte quality. We found that ARD affects gametes’ reproductive capacity and prevents oogenic germ cells from undergoing prolonged arrest in diakinesis. During ARD, germ cell apoptosis is very active; however, in contrast to a previous report, we found that it is not important for gonad shrinking. We observed that increased germ cell apoptosis during ARD depends partially on *lin-35*/Rb and an unknown mechanism. We propose that ovulation causes gonad shrinking by exhausting gonad contents when a few oocytes are produced. Finally, DAF-2 inactivation causes gonad shrinking in the presence of food suggesting that it may partially participate in this pathway. Here, we describe the effects of ARD on fertility and the regulation of germ cell apoptosis under starvation conditions.

## Materials and methods

### Strains

*C*. *elegans* strains were maintained as described previously [11]. All strains were grown at 20°C or the permissive temperature using *Escherichia coli* OP50 as food. The wild-type strain was N2 Bristol. For the *daf-2* experiments, 15°C and 25°C were used as the permissive and restrictive temperatures, respectively. For the *pha-4(zu225)* experiments, 24°C and 15°C were used as the permissive and restrictive temperatures, respectively. Heterozygous EU31 *skn-1(zu135)* animals segregate as Unc and WT: Unc individuals were picked for maintenance, and WT individuals laid eggs that did not hatch. For the *fog-1(q253)* experiments, 15°C and 25°C were used as the permissive and restrictive temperatures, respectively. *fog-1(q253)* worms were maintained at 15°C and upshifted to 25°C to feminize their germline. The alleles used were as follows: JK560 *fog-1(q253)*, CB4108 *fog-2(q71)*, MD701 *ced-1*::*gfp(bcIs39)*, *cep-1(gk138);ced-1*::*gfp(bcIs39)*, MT3002 *ced-3(n1286)*, RB2071 *ced-3(ok2734)*, MT1522 *ced-3(n717)*, CB1370 *daf-2(e1370)*, DR1309 *daf-16(m26);daf-2(e1370)*, MT10430 *lin-35(n745)*, GR1307 *daf-16(mgDf50)*, EU31 *skn-1(zu135)*, SM190 *pha-4(zu225)*, VC446 *alg-1(gk214)*, RB1206 *rsks-1(ok1255)*, SS712 *ife-1(bn127)*, WS2973 *gla-3(op212)*, RN083 *daf-2(e1370);ced-1*::*gfp*, and RN084 *lin-35(n745);ced-1*::*gfp*. All the strains were obtained from the *Caenorhabditis* Genetics Center (CGC).

### Image acquisition

Animals were mounted with 10 μl of 0.01% tetramisole in M9 on 2% agarose pads and observed using a Nikon Eclipse E600 microscope equipped with an AxioCam MRc camera (Zeiss). Images were obtained using Axio Vision software (Zeiss) and processed with ImageJ software.

### Starvation protocol

We performed the starvation protocol described by Seidel and Kimble (2011) [8]. Synchronous L1 populations were obtained by bleaching gravid hermaphrodites. The resulting embryos were washed in M9, transferred to conical tubes and incubated for 18 h at 20°C in a gyratory rocker. Hatched L1s were collected and placed in 10 cm plates seeded with OP50 at a density of ~1,200 L1s per plate until they reached the mid-L4 stage. To initiate starvation, the animals were collected with M9 medium and washed up to 6 times until no turbidity was observed; they were then placed in 10 cm plates with or without food at a density of ~10,000 animals per plate.

### Apoptosis assay

Cell corpses were counted using the MD701 *bcIs39* [*Plim-7*::*ced-1*::*gfp;lin-15(+)*] transgenic strain. After the starvation protocol, animals were placed in seeded plates as the control condition or in unseeded plates as the starvation condition. Animals were transferred daily until they ceased laying eggs and were then picked, anesthetized, mounted and visualized under an epifluorescence microscope.

### Germ cell quantification

We used DAPI staining to quantify the germ cells in each gonad arm as described by Silva-García and Navarro (2013) with some modifications [12]. Animals were dissected on glass coverslips, which were then inverted and placed on a polylysine-treated slide. The samples were freeze-cracked, fixed, stained with DAPI, mounted using 10 μl of Vectashield (Vector Lab) and visualized through fluorescence microscopy.

### Fertility assay

We determined the brood size and embryonic lethality, resulting from self-fertilization and mating as described by Bukhari *et al*., 2012 [13]. Single mid-L4 hermaphrodites were transferred to seeded NGM plates and maintained at 20°C. Animals were transferred to fresh plates each day until they ceased laying eggs. For the recovery of self-fertilizing animals following starvation, single hermaphrodites that had spent 5 days under starvation were transferred to seeded NGM plates and then to fresh plates each day until they ceased laying eggs. For mating animals, either mid-L4, well-fed 6-day-old or recovered *fog-(q253)* or *fog-2(q71)* animals were individually transferred to seeded NGM plates and crossed with 4 well-fed 1-day-old wild-type males. Twenty-four hours after the transfer of hermaphrodites, the embryos that did not hatch were scored as dead embryos. Forty-eight hours after the transfer of hermaphrodites, the number of larvae was scored.

### Long-term immobilization

We determined the temporal progression of germ cell corpse clearance using a method described by Kim *et al*., 2013 [14]. Briefly, animals were immobilized using 0.5 μl of a suspension of polystyrene beads (Polysciences, 2.5% by volume, 0.1 μm diameter) on 10% agarose pads. Germ cell corpses were visualized and imaged at 60X magnification until they were completely cleared.

### Ovulation rate

We followed the methodology described by Huang *et al*., 2012 [15]. Animals were individually scored for the number of embryos within the uterus using Nomarski microscopy, then transferred to fresh plates with food for 4 h and finally scored for the number of embryos within the uterus and those laid on the plate. More than 30 animals were observed for each genotype. The ovulation rate per gonad arm per hour = (embryos at the end of a time interval—embryos at the beginning) / (2 x number of animals x time interval).

### Statistical comparisons

The t-test or Mann-Whitney U test was used for comparisons with controls in fertility assays. For multiple comparisons in apoptosis, fertility assays and germ cell corpse clearance assays, the data were analyzed using one way ANOVA and Dunn’s test for multiple comparisons.

## Results

### Animals that undergo ARD do not recover their full fertility because this condition affects germ cell quality

**Fig 2:**
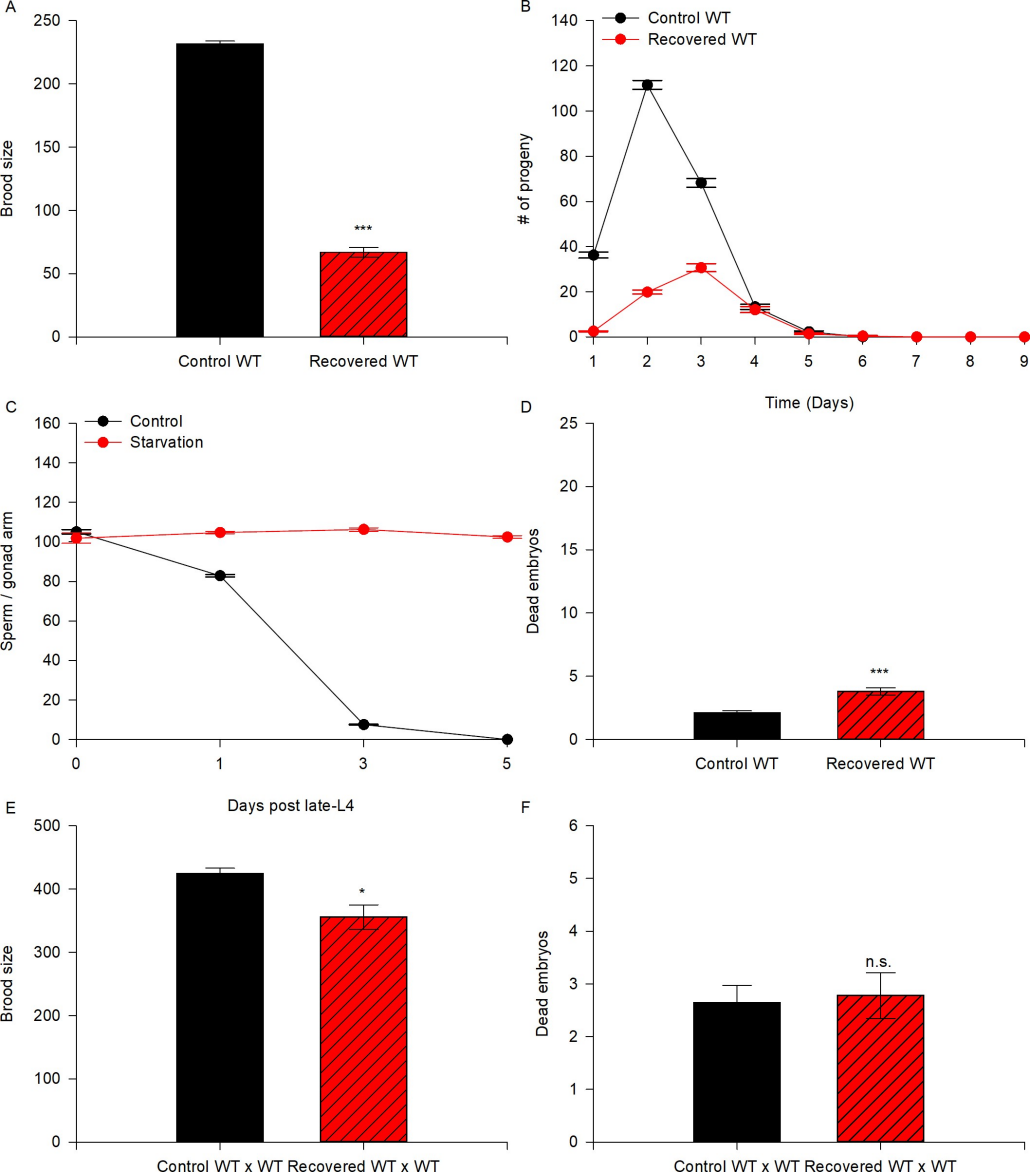
Animals subjected to ARD do not recover their fertility due to defects in germ cells. (A) The graph represents the brood size produced by self-fertilizing control (black) and recovered (red) wild-type animals. Mid-L4 hermaphrodites were allowed to self-fertilize (black) or were starved for 5 days and then refed (red). The data represent the mean brood size (±SEM) per animal. Statistical significance was determined by the Student’s t-test (P ≤ 0.001). (B) Quantification of the progeny produced on each day by self-fertilizing control (black line) and recovered (red line) wild-type animals. Time-course data are displayed as the mean (±SEM) number of progeny per time point. (C) Quantification of sperm produced by wild-type animals under control conditions (black line) or subjected to ARD (red line) at several time points: the late-L4 larval stage and 1, 3 and 5 days after the mid-L4 larval stage. The data are displayed as the mean (±SEM) number of sperm per time point. (D) The number of dead embryos within the progeny of self-fertilizing wild-type animals under control (black) and recovered conditions (red) was calculated. Data represent the mean number of dead embryos (±SEM) per animal. Statistical significance was determined by the Mann-Whitney rank sum test (P ≤ 0.001). (E) The graph represents the brood size produced by mating control (black) and recovered (red) wild-type animals. Well-fed mid-L4 hermaphrodites were individually mated to 4 well-fed wild-type males overnight and were then transferred individually to fresh plates until they ceased laying eggs (black). For the recovered animals, mid-L4 hermaphrodites were starved for 5 days, then recovered on food and mated to 4 well-fed 1-day-old wild-type males overnight, then transferred individually to fresh plates until they ceased laying eggs (red). The data represent the mean brood size (±SEM) per animal. Statistical significance was determined by the Mann-Whitney rank sum test (P ≤ 0.024). (F) The number of dead embryos within the progeny produced by mating wild-type animals under control (black) and recovered conditions (red) to well-fed wild-type males was calculated. Data represent the mean number of dead embryos (±SEM) per animal. Statistical significance was determined by the Mann-Whitney rank sum test, and the difference was not significant (n.s.).

We wanted to study how fertility and germ cell quality are affected after exposing animals to ARD. Although it has been reported that wild-type animals survive up to 30 days in ARD, their self-fertility is severely impaired after 15 days of starvation [7]. Therefore, we decided to study the effect of ARD when animals are exposed to 5 days of starvation because this period is sufficient to reduce hermaphrodite self-fertility by up to 50% [7]. To conduct our experiments, we used synchronized populations of mid-L4 larvae that were fed (control) or starved (no bacteria) for 5 days and then refed. For simplicity we will refer to starved/refed nematodes as recovered animals. We quantified the brood size of self-fertilizing recovered wild-type hermaphrodites and compared it to that of well-fed nematodes. We found that the recovered wild-type hermaphrodites produced smaller broods than those that grew under control conditions (29% of the wild-type brood size; Table 1, Fig 2A).

**Table 1:** Fertility assays in diverse genetic backgrounds used in this study.

Genotype	Brood Size				Dead Embryos				N	
WT	231.8	±	2.3		2.1	±	0.1		144	
Recovered WT	67.0	±	2.9	***	3.8	±	0.3	***	69	
Control WT	424.9	±	8.3		2.6	±	0.3		22	
x WT males										
Recovered WT	344.8	±	26.3	n.s	2.8	±	0.6	n.s.	17	
x WT males										
Control *fog-2(q71)*	324.5	±	6.5		2.4	±	0.3		46	
x WT males										
Recovered *fog-2(q71)*	183.2	±	5.6	*	4.0	±	0.3	*	42	
x WT males										
Prolonged diakinesis *fog-2(q71)*	55.8	±	2.3	*	4.8	±	0.2	*	51	
x WT males										
Control *fog-1(q253)*	302.3	±	7.0		2.5	±	0.1		31	
x WT males										
Recovered *fog-1(q253)*	177.1	±	3.3	*	4.4	±	0.2	*	35	
x WT males										
Prolonged diakinesis *fog-1(q253)*	65.0	±	1.6	*	5.6	±	0.1	*	39	
x WT males										
Control *ced-3(n717)*	141.0	±	2.6		8.1	±	0.7		43	
Recovered *ced-3(n717)*	53.1	±	1.8	*	7.3	±	0.5	*	41	
Control *ced-3(n1286)*	143.8	±	3.2		7.5	±	0.4		88	
Recovered *ced-3(n1286)*	52.0	±	2.3	*	12.3	±	0.8	*	58	
Control *ced-3(n717)*	315.6	±	8.6		19.8	±	3.0		11	
x WT males										
Recovered *ced-3(n717)*	248.9	±	12.3	**	19.3	±	1.9	n.s.	20	
x WT males										

Hermaphrodites with the different genetic backgrounds were individually selected at the mid-L4 stage and transferred to new plates every 24 h until they ceased laying embryos. Recovered wild-type hermaphrodites were selected at the mid-L4 stage, starved for 5 days and transferred to new plates every 24 h until they ceased laying embryos. t-test (control vs. recovered). Control *fog-1(q253)* and *fog-2(q71)* animals were individually selected in the mid-L4 stage, mated with well-fed males and transferred to fresh plates daily until they ceased laying embryos. Recovered virgin *fog-1(q253)* and *fog-2(q71)* animals were selected in the mid-L4 stage and starved for 5 days, then individually refed for 1 day, mated with well-fed males and transferred to fresh plates daily until they ceased laying embryos. Virgin *fog-1(q253)* and *fog-2(q71)* animals were selected in the mid-L4 stage and placed on food for 6 days, then mated with well-fed males and transferred to fresh plates daily until they ceased laying embryos. Plates were scored for dead embryos and total progeny. Embryos that did not hatch within 24 h after being laid were scored as dead. Dunn’s test (wild-type values as control). n.s. non significant.

* P ≤ 0.05

** P ≤ 0.01

*** P≤ 0.001

When we analyzed the progeny produced by the control and recovered animals by day, we observed that recovered animals produced fewer offspring during the first two days after refeeding, after which progeny production peaked at the third day and ceased at the fifth day (Fig 2B). It is likely that the delay in offspring production in recovered animals was due to the gonad regeneration process, which usually takes two days.

One explanation for the low fertility after ARD is that because the starvation experiments started at the mid-L4 larval stage (approx. 4 h after L4 molting), when spermatogenesis has not yet been completed, insufficient sperm production could occur. To discard this possibility, we quantified sperm production in starved animals once they were close to completing the L4 larval stage (approx. 8 h after L4 molting) by DAPI staining. Control wild-type animals produced an average of 105.11± 1.1 sperm (N = 36, Fig 2C) and starved wild-type animals produced a similar number of 101.89 ± 2.5 (N = 37). After 5 days of L4 larval molting, the control wild-type animals did not have any more sperm in their spermatheca, while 5-day-starved wild-type animals still harbored an average of 102.5 ± 0.68 sperm (N = 28, Fig 2C). We conclude that even during prolonged starvation sufficient sperm are produced, therefore this is not a factor that explains low progeny numbers after ARD.

We also quantified the embryonic lethality of self-fertilizing recovered hermaphrodites after ARD and found that recovered animals exhibited significantly higher embryonic lethality than control animals (3.8 ± 0.3 dead embryos/worm in recovered animals vs. 2.1 ± 0.1 dead embryos/worm in control animals, i.e., 1.8-fold; Table 1, Fig 2D).

To counteract the effect of ARD on sperm, we quantified the brood size and embryonic lethality of wild-type hermaphrodites that were exposed to ARD and later crossed with well-fed wild-type males. Under control conditions, mid-L4 wild-type hermaphrodites were crossed with 4 wild-type males overnight and then transferred daily to new Petri dishes until they ceased laying embryos. For ARD, mid-L4 wild-type animals were deprived of bacteria for 5 days, then transferred to plates with food and immediately crossed with 4 well-fed wild-type males. We observed that the fertility of recovered wild-type animals crossed with well-fed males improved considerably (81% of that in control wild-type animals) even though their brood size never reached that of the control (Fig 2E). Additionally, we did not observe significant differences in embryonic lethality between these two groups of animals (2.6 ± 0.3 dead embryos in control mated animals vs. 2.8 ± 0.6 dead embryos in recovered mated animals; Table 1, Fig 2F). Our results suggest that ARD affects fertility and embryonic survival due to defects in oogenic germ cells; however, we were not able to rule out the possibility that sperm quality is impaired under starvation conditions.

### Feminized germlines are more sensitive to ARD than those of wild-type animals

**Fig 3:**
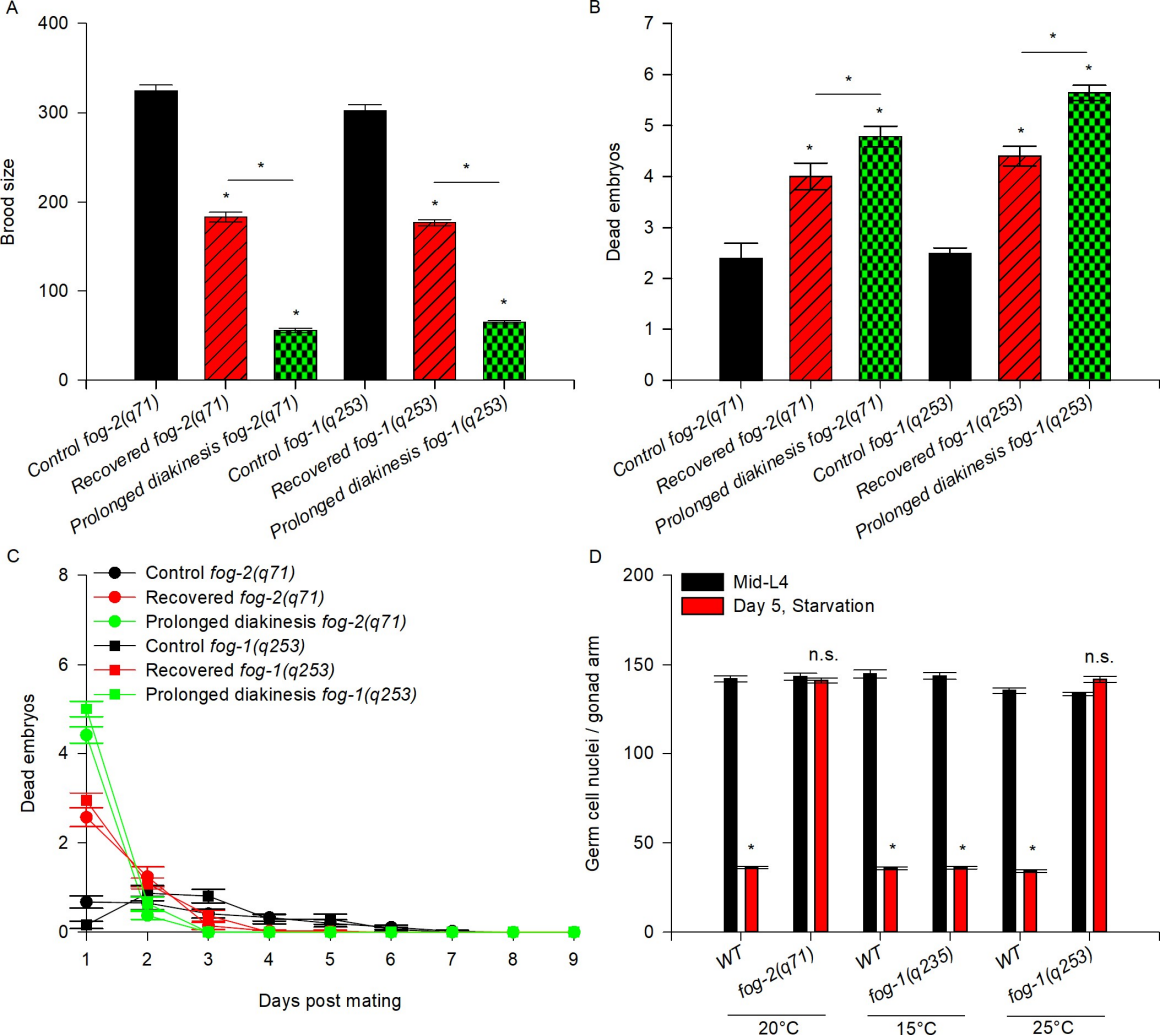
Germlines from feminized mutant backgrounds are more sensitive to ARD that those of wild-type animals. (A) The graph represents the brood size produced by mating virgin *fog-2(q71)* and *fog-1(q253)* mutant animals to well-fed wild-type males. Mid-L4 virgin animals from different genetic backgrounds were mated to 4 well-fed wild-type males overnight, then transferred individually to fresh plates until they ceased laying eggs (black). Mid-L4 virgin mutant animals were starved for 5 days, recovered on food for 24 h and then mated with 4 well-fed 1-day-old wild-type males overnight, after which they were transferred individually to fresh plates until they ceased laying eggs (red). Well-fed 6-day-old virgin mutant animals, which exhibited stacked oocytes arrested in prolonged diakinesis within the gonad, were mated with 4 well-fed 1-day-old wild-type males overnight and then transferred individually to fresh plates until they ceased laying eggs (green). The data represent the mean brood size (±SEM) per animal. Statistical significance was determined by one-way ANOVA on ranks, followed by Dunn’s test (P<0.05). (B) The graph shows the number of dead embryos within the progeny produced by mating with well-fed wild-type males under control conditions (black), recovered conditions (red) and prolonged arrest in diakinesis (green). The data represent the mean number of dead embryos (±SEM) per animal. Statistical significance was determined by one-way ANOVA on ranks, followed by Dunn’s test (P<0.05). (C) Quantification of dead embryos within the progeny produced each day by mating with wild-type males under control (black line) and recovered (red line) conditions and prolonged arrest in diakinesis (green line). Time course data are displayed as the mean (±SEM) number of dead embryos per time point. (D) Number of germ cells per gonad arm in wild-type and *fog-2 (q71)* and *fog-1(q253)* mutant animals. The graph represents the average number of germ cells scored using DAPI staining in dissected gonads during the mid-L4 stage (0) and 5 days after the mid-L4 stage under starvation conditions.

To test the effect of ARD exclusively on oogenic germ cells, we used *fog-1(q253)* and *fog-2(q71)* mutant animals, which have feminized germlines. Female *fog-2(q71)* animals are unable to produce sperm and only reproduce when they are crossed with males [16]. *fog-1(q253)* hermaphrodites are temperature sensitive and at the restrictive temperature (25°C) they exhibit feminized gonads [17]. Under control conditions, single mid-L4 virgin *fog-2(q71)* females were crossed with 4 wild-type males overnight and then transferred daily to new Petri dishes until they ceased laying embryos. For ARD, virgin mid-L4 *fog-2(q71)* females were deprived of bacteria for 5 days, then recovered on food for 24 h and crossed with 4 wild-type males overnight. We compared the brood size and embryonic lethality of control and recovered *fog-2* mutant animals. We observed that recovered *fog-2(q71)* animals produced smaller broods than control *fog-2(q71)* animals (56.5% of the brood size of control animals, Table 1, Fig 3A) and showed higher embryonic lethality (1.7-fold; 2.4 ± 0.3 dead embryos/worm in *fog-2* control animals vs. 4 ± 0.3 dead embryos/worm in *fog-2* recovered animals; Table 1, Fig 3B).

To confirm our findings we compared the brood size and embryonic lethality of control and recovered *fog-1(q253)* mutant animals. To do so, a group of synchronized hermaphrodite *fog-1(q253)* animals were grown from L1-mid-L4 at 25°C and then individually transferred to a plate with bacteria and 4 wild-type males overnight as a control. Another group of *fog-1(q253)* animals was transferred to plates without bacteria and incubated for 5 days at 25°C. On the fifth day, the animals were recovered for 24 h in a plate with bacteria and then individually transferred to plates with bacteria and crossed with 4 wild-type males overnight. Recovered *fog-1(q253)* mutant animals showed significantly fewer progeny than control animals (Fig 3A and Table 1). Additionally, recovered *fog-1(q253)* mutant animals showed higher embryonic lethality (1.7-fold; 2.5 ± 0.1 dead embryos/worm in *fog-1* control animals vs. 4.4 ± 0.2 dead embryos/worm in *fog-1* recovered animals; Table 1, Fig 3B). Our results demonstrate that ARD affects oogenic germ cells to an extent that could interfere with embryo survival long after starvation has been ended. Since we observed that the effect of ARD was stronger in feminized mutants, we conclude that some genetic backgrounds could be more sensitive than others.

### Oogenic germline starvation response prevents germ cells from entering oogenesis arrest

**Fig 4:**
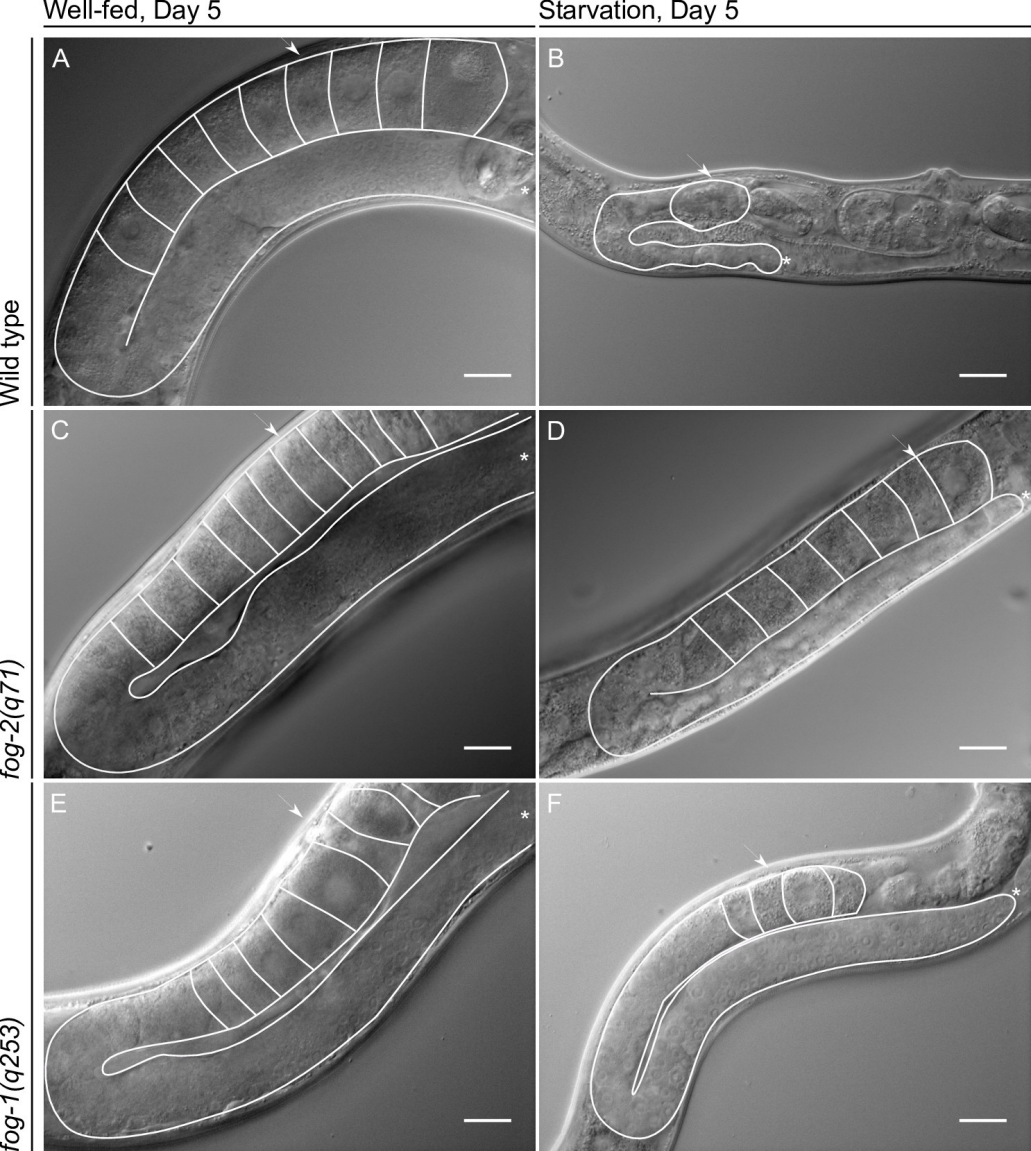
The gonads of feminized germline mutant animals do not shrink during ARD. Nomarski gonad images of the indicated genetic backgrounds and conditions. (A) Representative Nomarski image of a well-fed 5-day-old wild-type hermaphrodite. (B) Nomarski image of a wild-type hermaphrodite that had spent 5 days in ARD. (C) A well-fed 5-day-old virgin *fog-2(q71)* mutant animal. (D) A virgin *fog-2(q71)* animal that had been starved for 5 days. (E) A well-fed 5-day-old virgin *fog-1(q253)* mutant animal. (F) A virgin *fog-1(q253)* animal that had been starved for 5 days. In all images one gonad arm and the oocytes within it are outlined in white; the distal gonad is marked with an asterisk (*); the arrow points to the proximal gonad. Scale bar = 20 μm.

Well-fed virgin *fog-2* mutant animals’ do not produce sperm; instead, all of their germ cells develop as oocytes, and the most proximal remain arrested in diakinesis within the gonad [16]. When *fog-2* mutant animals are mated after prolonged diakinesis arrest, their fertility and embryonic viability are severely affected [3]. By the fifth day after L4 molting, well-fed wild-type hermaphrodites have exhausted their sperm supply and arrested oocytes in diakinesis can be observed in the gonad (approx. 9) (Fig 4A). During the oogenic germline starvation response in wild-type animals, oogenesis is delayed but does not seem to be arrested (at least for the first 3 days after fasting); no stacking oocytes are observed and it is possible to distinguish only one developing oocyte per gonad arm ([8] and Fig 4B). In contrast, we observed stacked oocytes in 5-day-old ARD virgin *fog-2* mutant animals (approx. 7, Fig 4D) although they exhibited fewer stacked oocytes than well-fed 5-day-old virgin *fog-2* animals (approx. 11, Fig 4C). We also observed that *fog-1(q253)* mutant animals that were starved for 5 days at the restrictive temperature showed stacked oocytes within their gonads (approx. 4, Fig 4F) although they presented fewer stacked oocytes than well-fed 5-day-old virgin *fog-1* animals (approx. 8, Fig 4E).

To continue testing whether the oogenic germline starvation response during ARD exerts a protective effect on germ cells, we compared the quality of germ cells when they were exposed to prolonged arrest in meiosis under well-fed conditions vs. ARD. We crossed well-fed 6-day-old virgin *fog-2* and *fog-1* mutant animals with well-fed wild-type males and determined their brood size and embryonic lethality, which were compared to those of *fog-2(q71)* and *fog-1(q253)* mutant animals crossed under control and recovered conditions. We found that well-fed 6-day-old *fog-2* and *fog-1* mutant animals produced smaller broods (by 17% and 22%, respectively) than the controls and even smaller broods (by 30.5% and 36.7%, respectively) than animals recovered after 5 days of ARD (Fig 3A, Table 1).

Additionally, well-fed 6-day-old *fog-2* and *fog-1* mutant animals exhibited higher embryonic lethality than control (by 2- and 2.2-fold, respectively) and recovered animals (1.2- and 1.3-fold, respectively) (Fig 3B and Table 1). We observed that dead embryos were present mainly during the first day in well-fed 6-day-old *fog-2* and *fog-1* mutant animals (Fig 3C). Similarly, in *fog-2* and *fog-1* mutant recovered animals, the main peak of dead embryos was observed on the first day after the cross (Fig 3C). Apparently, the dead embryos were produced from the oocytes that were arrested in diakinesis for the longest period during ARD. We observed that embryonic lethality in *fog-2(q71)* and *fog-1(q253)* mutant animals was mainly caused by embryos that resulted from the fertilization of the oocytes that were already present or produced during ARD and presumably were arrested for a long period of time. We conclude that because the oogenic germline starvation response during ARD slows oocyte production, oogenic germ cells are prevented from undergoing diakinesis arrest, which preserves oocyte quality. We suggest that this could explain why feminized germline mutant animals are more sensitive to ARD than wild-type animals.

During the course of these experiments, we observed that the gonads of *fog-2* and *fog-1* animals did not shrink during ARD (Fig 4D and 4F). Our results, as well as those of other similar approaches reported previously [18], show that feminized mutant animals’ gonads do not decrease in size, suggesting that ovulation is one of the causes of gonad shrinking during ARD. To verify that feminized gonads do not shrink during ARD, we quantified the germ cell number per gonad arm using DAPI staining in control animals and in animals that spent 5 days in ARD. In well-fed conditions, wild-type animals showed an increase in the number of germ cells per gonad arm from 141.9 ± 1.8 (at mid-L4) to 468.23 ± 2.0 (at the fifth day post mid-L4) (Table 2). Additionally, the number of germ cells per gonad arm decreased progressively during starvation from 141.9 ± 1.8 (at the mid-L4) to 36.0 ± 0.5 (at the fifth day post mid-L4) (Fig 3D and Table 2). We found that the number of germ cells in *fog-2* mutant animals that were starved for 5 days remained unchanged compared to the number of germ cells per gonad arm at the mid-L4 stage (143.1 ± 2.0 vs. 141.0 ± 1.5; Fig 3D and Table 2). We confirmed this result using *fog-1(q253)* mutant animals (Fig 3D and Table 2). Moreover, when *fog-1(q253)* animals were maintained at the permissive temperature (15°C) from L1 to adulthood and subjected to ARD, their gonads were able to shrink similarly to those of the wild-type (Fig 3D and Table 2). Our results suggested that gonad shrinking during ARD is partially due to ovulation.

**Table 2:** Germ cell quantification per gonad arm in different genetic backgrounds under control and starvation conditions.

							* *			5 Days post mid-L4							
		mid-L4			N		Control			N		Starvation			N		
	WT	141.9	±	1.8	49		468.2	±	2.0	38	**	36.0	±	0.5	54	**	
20°C																	
	*fog-2(q71)*	143.1	±	2.0	42		315.9	±	2.6	47	**	141.0	±	1.5	48	**	
	WT	144.7	±	2.2	30		386.9	±	1.6	20	**	35.5	±	0.8	24	**	
15°C																	
* *	*fog-1(q253)*	143.5	±	1.8	30		386.2	±	1.6	30	**	36.0	±	0.6	25	**	
	WT	135.3	±	1.5	20		388.5	±	1.8	25	**	34.2	±	0.7	25	**	
25°C																	
* *	*fog-1(q253)*	133.5	±	0.8	30		338.5	±	1.8	30	**	141.8	±	1.6	25	n.s.	

The gonads of animals under control conditions or starvation were dissected, stained and scored for the number of germ cells using epifluorescence microscopy. Control animals remained on NGM plates seeded with OP50. For starvation conditions, animals were grown on food from L1 to the mid-L4 larval stage and then starved for 5 days. Animals were subsequently picked and dissected. The dissected gonads were stained with DAPI and the number of germ cells per gonad arm was scored under fluorescence microscopy.

### Germ cell apoptosis is not required to reduce gonad size during prolonged starvation

**Fig 5:**
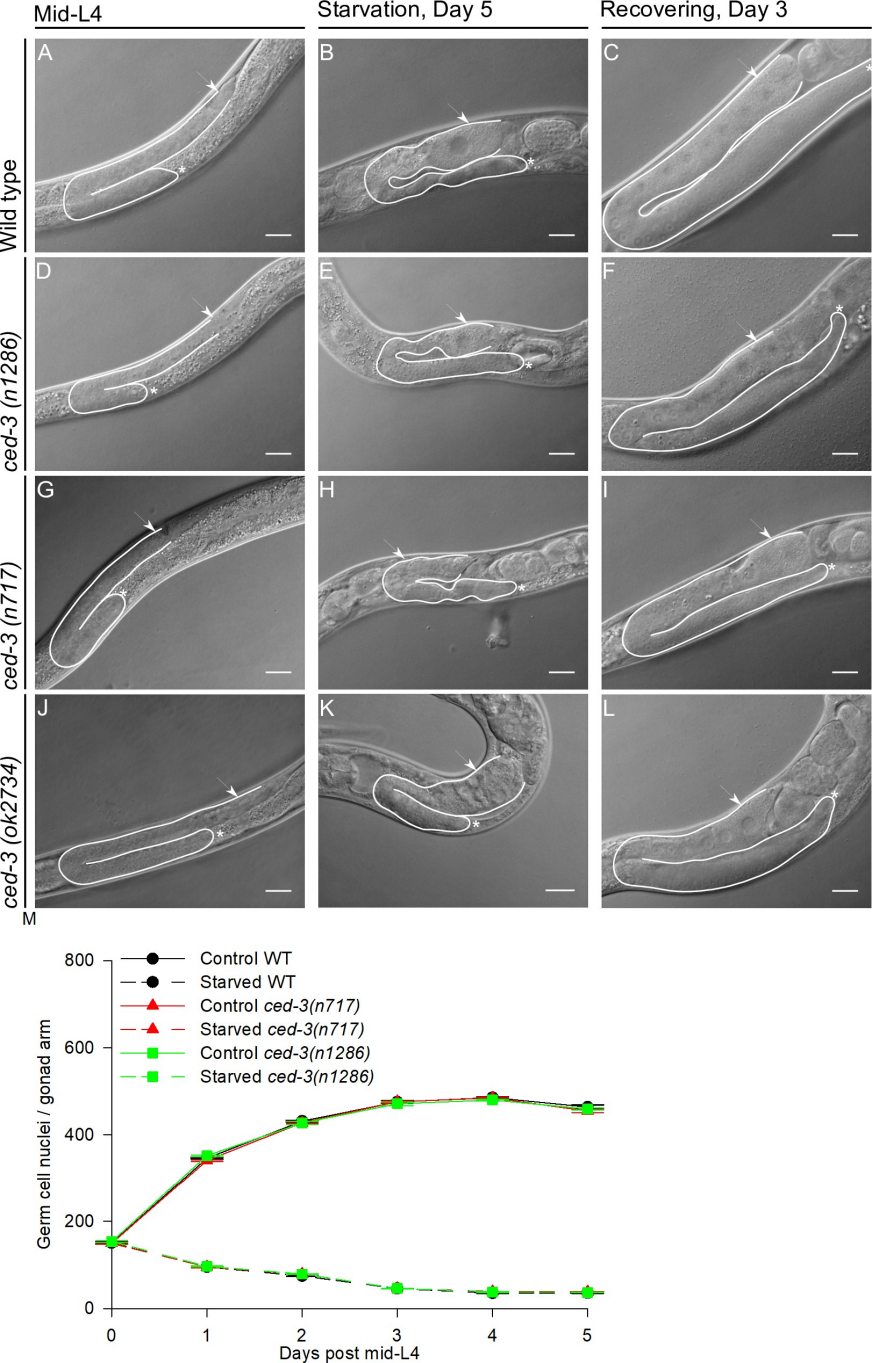
Apoptosis is not required to reduce gonad size during the oogenic germline starvation response. Nomarski images of gonads of mid-L4 wild-type animal and animals with the three different alleles of *ced-3* (A, D, G and J). Nomarski images of gonads of wild-type and *ced-3* mutant animals starved for 5 days (B, E, H and K). Nomarski images of gonads of wild-type and *ced-3* animals that spent 5 days under starvation and were recovered on food for 3 days (C, F, I and L). In all images one gonad arm is outlined in white; the distal gonad is marked with an asterisk (*); the arrow points to the proximal gonad. Scale bar = 20 μm. (M) Germ cell nuclei per gonad arm scored in wild-type animals and two *ced-3* defective mutants by DAPI staining. The graph shows the average number of germ cell nuclei per gonad arm in control conditions (solid lines) and during starvation (dotted lines) ±SEM at each point.

Caspase CED-3 is required for germ cell apoptosis under control and starvation conditions [19, 20]. Angelo and van Gilst, (2009) previously showed that during ARD, the gonads of *ced-3(n1286)* mutant animals are not able to shrink and they suggested that apoptosis is required for this process [7]. However, we obtained different results. We exposed mid-L4 *ced-3* mutant animals (alleles *n1286*, *n717* or *ok2734)* to prolonged-starvation and we observed that the gonads of animals harboring all of these alleles were able to shrink during fasting (Fig 5D, 5E, 5G, 5H, 5J and 5K) similarly to those of the wild-type (Fig 5A and 5B).

To verify that the gonads of *ced-3* mutant animals shrink similarly to those of wild-type animals, we quantified the number of germ cells per gonad arm by DAPI staining from the mid-L4 stage over the next 5 days under control and starvation conditions in animals with the wild-type and *ced-3(n717)* and *(1286)* mutant alleles. We found that *ced-3* mutant animals indeed showed a progressive reduction of the germ cell number per gonad arm during starvation similar to that in wild-type animals (Fig 5M and Table 3). Moreover, the gonad size of *ced-3* mutant animals recovered when they were refed as efficiently as that of wild-type animals, as judged by Nomarski microscopy (Fig 5C, 5F, 5I and 5L) and DAPI staining (Table 3). Our data demonstrate that germ cell apoptosis is not required for gonad shrinking during prolonged starvation or gonad regeneration after fasting.

**Table 3:** Germ cells per gonad arm under control, starvation and recovered conditions.

		WT			* *								
Days post mid-L4		Control			N	Starvation			N	Recovering			N
0		150.4	±	2.5	36	150.4	±	2.5	36	339.2	±	3.5	25
1		346.1	±	2.0	35	95.1	±	1.0	36				
2		431.3	±	1.9	35	74.5	±	0.9	36				
3		475.6	±	2.7	25	45.6	±	0.7	35				
4		485.3	±	1.4	30	35.0	±	0.6	36				
5		463.7	±	4.7	24	35.5	±	0.9	35				
		*ced-3(n717)*			* *								
Days post mid-L4		Control			N	Starvation			N	Recovering			N
													
0		145.5	±	4.6	35	145.5	±	4.6	35	342.9	±	2.8	30
1		340.9	±	1.9	36	93.7	±	1.9	35				
2		427.8	±	1.7	36	78.9	±	1.2	35				
3		475.8	±	2.7	24	46.1	±	0.8	35				
4		484.6	±	2.4	25	38.6	±	0.8	36				
5		454.7	±	3.7	25	38.1	±	0.8	35				
		*ced-3(n1286)*			* *								
Days post mid-L4		Control			N	Starvation			N	Recovering			N
													
0		153.7	±	2.1	36	153.7	±	2.1	36	342.6	±	2.7	30
1		351.8	±	1.8	25	96.8	±	1.3	35				
2		427.1	±	3.3	26	78.8	±	0.9	36				
3		471.2	±	3.9	25	46.1	±	0.6	35				
4		479.7	±	2.2	25	37.7	±	1.0	36				
5		459.2	±	2.9	25	36.7	±	0.8	35				

The gonads of animals under control conditions, starvation and recovery were dissected, stained and scored for the number of germ cells using epifluorescence microscopy. Control animals were placed in NGM plates seeded with OP50 and transferred to fresh plates daily until they ceased laying eggs. For starvation conditions, animals were placed in NGM plates unseeded and transferred to fresh plates daily, similar to the control animals. Animals were picked and dissected. The dissected gonads were stained with DAPI, and the number of germ cells per gonad arm was scored under fluorescence microscopy. For recovery, animals that had spent 5 days in ARD were transferred to plates seeded with OP50 for 3 days. Animals were then picked and dissected.

Angelo and van Gilst (2009) also reported that *ced-3(n1286)* caspase mutant animals were unable to produce progeny after 15 days of prolonged starvation and suggested that apoptosis was essential to maintain fertility after fasting [7]. We observed that neither wild-type nor *ced-3(n717 and n1286)* mutant animals produced any progeny after spending 15 days in ARD (N = 37, 39 and 42, respectively), even after they were crossed with wild-type males (N = 27, 29 and 30, respectively).

**Fig 6:**
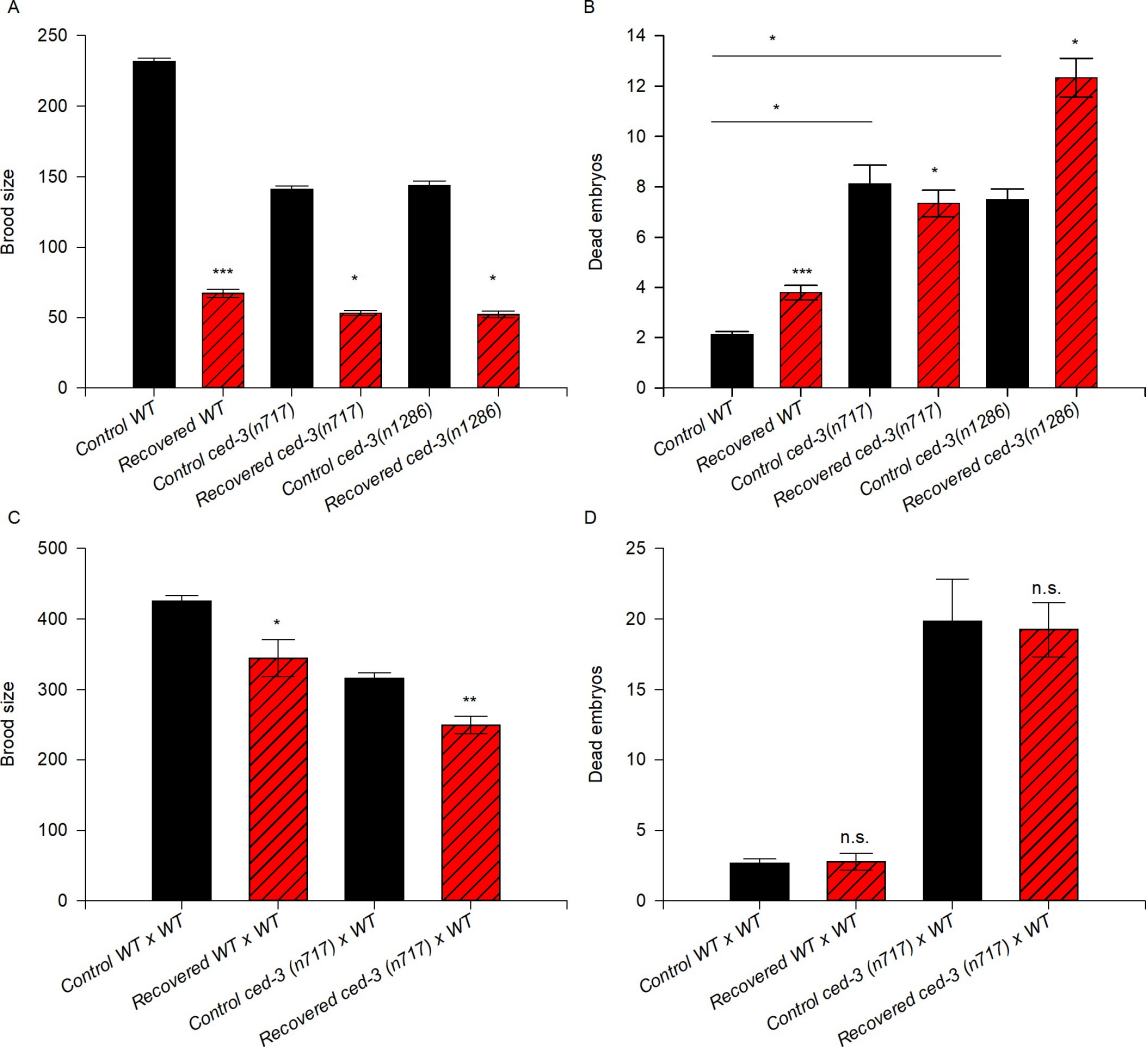
Apoptosis is as important to preserve oocyte quality after ARD as in control conditions but it is not essential for recovering fertility after ARD. (A) The graph represents the brood size produced by control (black) and recovered (red) wild-type and *ced-3* mutant animals. Mid-L4 hermaphrodites of the different genetic backgrounds were allowed to self-fertilize (black) or were starved for 5 days and then refed (red). The data represent the mean brood size (±SEM) per animal. Statistical significance was determined by the t-test (WT control vs. recovered) or by ANOVA on ranks and Dunn’s test for multiple comparisons. (B) The number of dead embryos within the progeny of self-fertilizing wild-type and *ced-3* mutant animals under control (black) and recovered conditions (red) was calculated. The data represent the mean number of dead embryos (±SEM) per animal. Statistical significance was determined by the Mann-Whitney rank sum test (P ≤ 0.001) (WT control vs. recovered) or by ANOVA on ranks and Dunn’s test for multiple comparisons. (C) The graph represents the brood size produced by mating control (black) and recovered (red) animals of the different genetic backgrounds. Well-fed mid-L4 hermaphrodites were individually mated with 4 well-fed wild-type males overnight, then transferred individually to fresh plates until they ceased laying eggs (black). For the recovered animals, mid-L4 hermaphrodites were starved for 5 days, then recovered on food and immediately mated with 4 well-fed 1-day-old wild-type males overnight and transferred individually to fresh plates until they ceased laying eggs (red). The data represent the mean brood size (±SEM) per animal. Statistical significance was determined by the Mann-Whitney rank sum test (P ≤ 0.024) in wild-type populations and by the Student’s t-test (P ≤ 0.001) in *ced-3(n717)* populations. (D) The number of dead embryos within the progeny produced by mating animals of the different genetic backgrounds under control (black) and recovered conditions (red) to well-fed wild-type males was calculated. Data represent the mean number of dead embryos (±SEM) per animal. Statistical significance was determined by the Mann-Whitney rank sum test.

We quantified the progeny produced by well-fed and recovered *ced-3* animals (alleles *n717* and *n1286*) and compared the number to that in wild-type animals after spending 5 days in ARD. Andux and Ellis (2008) previously reported that *ced-3* mutant animals (with the *n718*, *n2439*, *n2921* alleles) produce fewer progeny and exhibit higher embryonic lethality than wild-type animals in control conditions [3]. Accordingly, we observed that the animals harboring the *ced-3* mutant alleles *n717* and *n1286* exhibited 40% fewer offspring on average than wild-type animals under control conditions (Fig 6A and Table 1). When we compared the number of progeny produced by recovered wild-type animals to that produced by recovered *ced-3* mutant animals, we observed that *ced-3* mutant animals produced 20–24% fewer progeny on average than recovered wild-type animals (Fig 6A and Table 1).

As reported by Andux and Ellis (2008), the embryonic lethality of *ced-3* mutant animals under normal conditions was higher than that of the wild-type (3.9-fold higher than the wild-type for the *ced-3(n717)* allele and 3.6-fold higher for *ced-3(n1286)*) ([3]; Fig 6B and Table 1). After ARD, *ced-3(n717)* mutant animals exhibited 1.9-fold higher embryonic lethality than recovered wild-type animals while *ced-3(n1286)* mutant animals presented 3.2-fold higher embryonic lethality than recovered wild-type animals (Fig 6B and Table 1).

To counteract the effect of ARD on sperm, we quantified the brood size and embryonic lethality of *ced-3(n717)* mutant animals exposed to ARD and subsequently crossed with well-fed wild-type males. We found that the fertility of recovered *ced-3(n717)* mutant animals crossed with well-fed males improved considerably (79% of that in the corresponding control) even though they did not reach the control brood size (Fig 6C). In control conditions, mated *ced-3(n717)* mutant animals exhibited higher embryonic lethality than mated wild-type animals (7-fold) but their embryonic lethality did not increase further after ARD (8-fold) (Fig 6D and Table 1). Our results confirmed that apoptosis is important to maintain oocyte quality under control conditions ([3] and this work). We also observed that apoptosis is important for fertility and to preserve oocyte quality during ARD; however, it is not essential, as previously claimed by Angelo and van Gilst (2009) [7].

### High germ cell apoptosis continues during ARD

**Fig 7:**
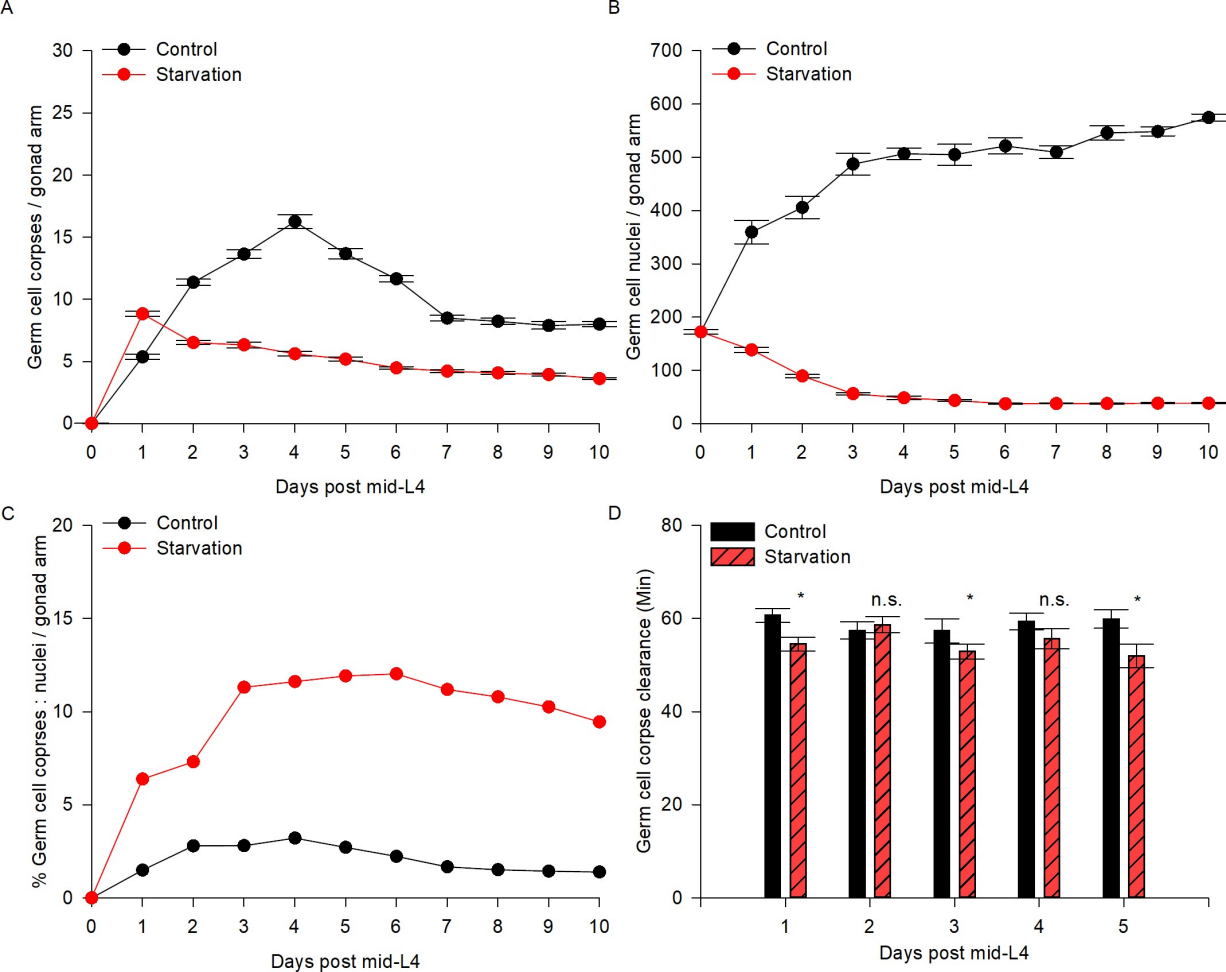
Germ cell apoptosis is increased and continues during ARD. **(**A) The *ced-1*::*gfp* transgene was used to visualize the number of germ cell corpses per gonad arm (see Materials and methods) for 10 days in control (black line) and starvation conditions (red line). The average number of germ cell corpses per gonad arm is shown with SEM. (B) DAPI staining was used to quantify the number of germ cells per gonad arm (see Materials and methods) for 10 days in control (black line) and starvation (red line) conditions. The average number of germ cells per gonad arm is shown with SEM. (C) The percentage of germ cell corpses among the total germ cells per gonad arm in fed animals (black line) and during starvation (red line) was calculated. (D) The time of germ cell corpse clearance was recorded every day after 1–5 days post-mid-L4 (see Materials and methods) in control (black bars) and starvation (red bars) conditions.

We investigated germ cell apoptosis dynamics during ARD. To quantify apoptosis, we used a germ cell apoptosis reporter *P*_*lim-7*_*ced-1*::*gfp*, which is expressed in the sheath cells and surrounding germ cell corpses [21]. In well-fed *ced-1*::*gfp* animals, the number of corpses per gonad arm increased with age and peaked at day 4 post-mid-L4 (16.3 ± 0.55 germ cell corpses per gonad arm), with an average of 9.65 ± 0.98 germ cell corpses per gonad over the following days (Table 4). After one day of starvation, we observed an increase of 1.6-fold in apoptosis compared to control conditions, with an average of 4.9 germ cell corpses per gonad arm over the following days (Fig 7A and Table 4).

**Table 4:** Germ cell apoptosis dynamics under control and starvation conditions.

	*ced-1*::*gfp*			* *					
Days post mid-L4	Control			N	Starvation				N
0	0.0	±	0.00	141	0.0	±	0.01	n.s.	140
1	5.4	±	0.21	139	8.8	±	0.20	**	140
2	11.4	±	0.26	129	6.5	±	0.15	**	141
3	13.6	±	0.34	111	6.3	±	0.24	**	129
4	16.3	±	0.55	103	5.6	±	0.18	**	123
5	13.7	±	0.40	111	5.2	±	0.16	**	143
6	11.6	±	0.26	121	4.5	±	0.10	**	135
7	8.5	±	0.22	110	4.2	±	0.11	**	129
8	8.2	±	0.25	119	4.1	±	0.10	**	144
9	7.9	±	0.30	76	3.9	±	0.11	**	124
10	8.0	±	0.21	72	3.6	±	0.08	**	105

The number of germ cell corpses was scored using *ced-1*::*gfp* animals under epifluorescence microscopy. Control mid-L4 hermaphrodites were placed in NGM plates seeded with OP50 and transferred to fresh plates daily until they ceased laying eggs; the animals were picked daily for up to 10 days and mounted with 20 μl of 0.01% tetramisole in M9 on 2% agarose pads. For starvation conditions, mid-L4 animals were placed in unseeded NGM plates and transferred to fresh plates daily similar to the control animals; the animals were picked daily for up to 10 days and mounted with 20 μl of 0.01% tetramisole in M9 on 2% agarose pads. n.s. non significant

* P ≤ 0.05

** P ≤ 0.01,.

It is remarkable that germ cell apoptosis continues and is very active during prolonged starvation particularly because germ cell proliferation stops after 30 minutes of fasting [9] and the gonad is completely shrunken after the fifth day. To quantify the proportion of germ cells that were being eliminated by apoptosis during starvation versus those that remained in the starved gonad, we determined the number of germ cells per gonad arm in dissected gonads under control and fasting conditions, using DAPI (4’,6’-diamino-2-phenylindole) staining. We found that under control conditions the number of germ cells per gonad arm increased gradually with age until day 4 post-mid-L4, after which the numbers of germ cells per gonad arm were similar (approx. 534.2 germ cells/gonad arm) (Fig 7B and Table 5). During prolonged-starvation, the germ cell number decreased gradually until reaching the minimal number at day 6 post-mid-L4, then remained almost unchanged over the course of the experiments (38.1 germ cells/gonad arm on average) (Fig 7B and Table 5).

**Table 5:** The number of germ cells per gonad arm under control and starvation conditions.

	*ced-1*::*gfp*			* *				
Days post mid-L4	Control			N	Starvation			N
0	172.2	±	4.57	45	172.2	±	4.57	45
1	359.6	±	21.88	15	138.4	±	4.72	42
2	406.1	±	21.23	15	89.4	±	3.04	30
3	487.3	±	20.82	17	56.0	±	1.83	31
4	506.8	±	10.94	15	48.4	±	3.02	27
5	505.1	±	19.53	18	43.6	±	1.37	35
6	521.3	±	14.82	18	37.2	±	1.18	30
7	509.6	±	11.76	16	37.8	±	0.75	24
8	545.9	±	13.44	17	37.8	±	1.04	38
9	548.6	±	8.19	17	38.4	±	1.19	34
10	574.7	±	6.56	15	38.4	±	1.19	38

The gonads of animals under control conditions and starvation were dissected, stained and scored for the number of germ cells using epifluorescence microscopy. Control animals were placed in NGM plates seeded with OP50 and transferred to fresh plates daily until they ceased laying eggs. For starvation conditions, animals were placed in unseeded NGM plates and transferred to fresh plates daily, similar to the control animals. Animals were picked and dissected. The dissected gonads were stained with DAPI and the number of germ cells per gonad arm was scored under fluorescence microscopy.

We quantified the percentage of germ cells eliminated by apoptosis (Table 4) and compared it to the number of germ cells present in the gonad (Table 5) under control conditions and during starvation. We found that under control conditions, approximately 1.91% of the total number of germ cells appeared as germ cell corpses at each time point. However, during starvation the percentage of germ cell corpses was considerably higher at each point compared to the control conditions (average of 9.27% of the total number of germ cells) (Fig 7C). Our data show that germ cell apoptosis increases up to 4.8-fold during the oogenic germline starvation response and continues under these conditions.

To test whether germ cell corpse clearance is affected during starvation, we quantified germ cell corpse engulfment in animals exposed to 1–5 days of starvation by time-lapse microscopy (see methods). We found that the timing of germ cell corpse clearance was not extended during the first 5 days of prolonged starvation compared to the control (approx. 55 minutes (prolonged starvation), similar to the control time of 58 minutes) (Fig 7D, Table 6). Our results demonstrate that the process of germ cell corpse engulfment remains active during prolonged starvation.

**Table 6:** Germ cell corpse clearance under control and starvation conditions.

		WT			* *					
Days post mid-L4		Control			N	Starvation				N
0		No corpses detected			25	No corpses detected				25
1		60.7	±	1.51	9	54.6	±	1.47	*	9
2		57.4	±	1.89	9	58.7	±	1.74	n.s.	9
3		57.3	±	2.63	9	52.9	±	1.61	*	9
4		59.4	±	1.74	8	55.6	±	2.15	n.s.	8
5		59.9	±	1.97	8	52.0	±	2.50	*	8

The timing of germ cell corpse clearance under control and starvation conditions was scored using a long-term immobilization technique and Nomarski microscopy at 60x magnification. Control mid-L4 hermaphrodites were placed in NGM plates seeded with OP50, picked daily for up to 5 days and immobilized with 0.5 μl of a polystyrene bead suspension on 10% agarose pads to observe an individual germ cell corpse and determine its clearance time in minutes. For starvation conditions, mid-L4 hermaphrodites were placed in unseeded NGM plates, picked daily for up to 5 days and then immobilized with 0.5 μl of a polystyrene bead suspension on 10% agarose pads to observe an individual germ cell corpse and determine its clearance time in minutes. The Holm-Sidak test for comparisons within groups was used. n.s. = non significant

* P < 0.05.

**Fig 8:**
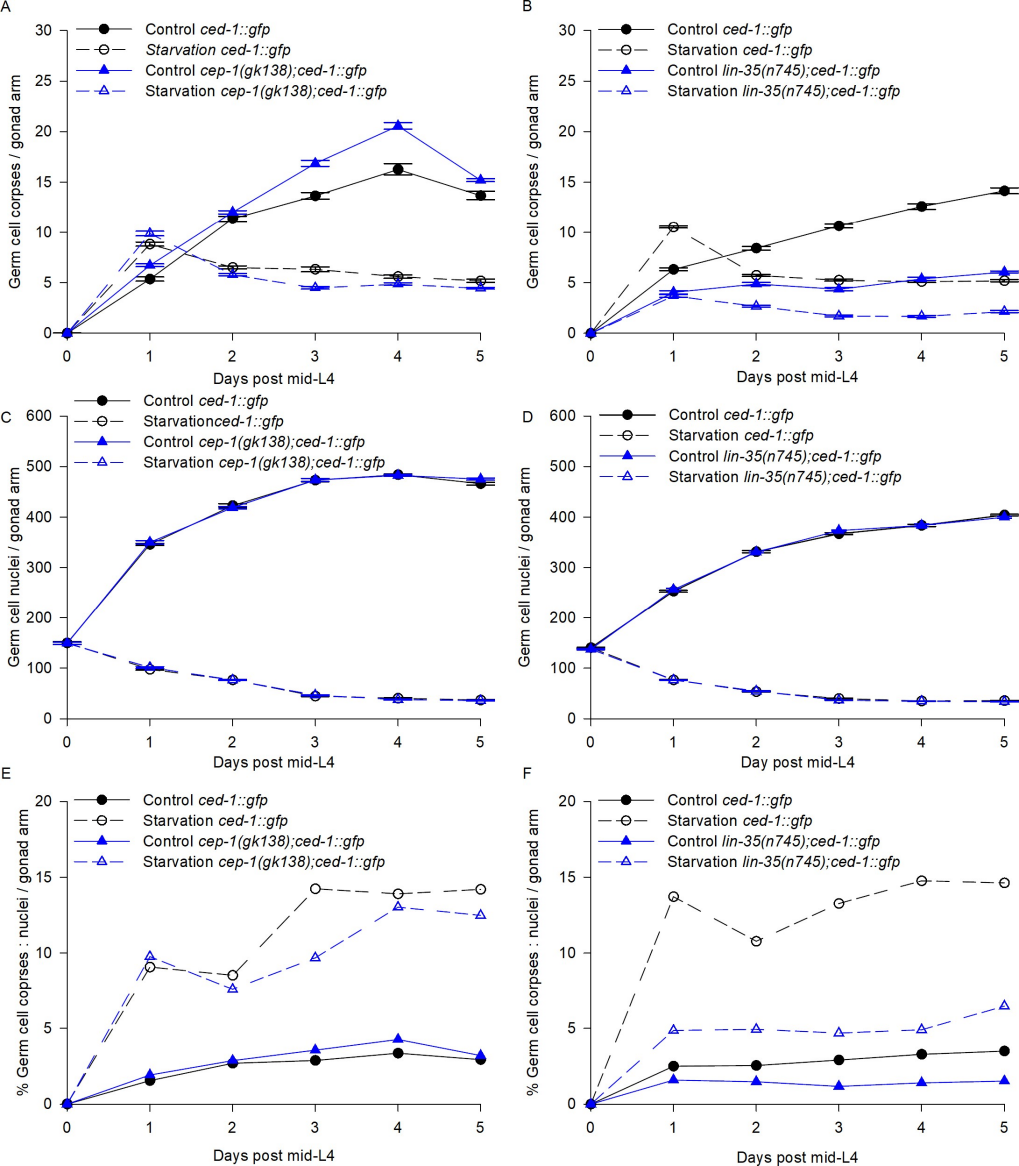
Germ cell apoptosis is p53 independent and partially regulated by *lin-35/Rb* during ARD. **(**A-B) *cep-1(gk38);ced-1*::*gfp*, *lin-35(n745);ced-1*::*gfp* and *ced-1*::*gfp* animals were used to visualize the number of germ cell corpses per gonad arm (see Materials and methods) for 5 days in control (solid lines) and starvation conditions (dotted lines). The average number of germ cell corpses per gonad arm is shown with the SEM. (C-D) DAPI staining was used to quantify the number of germ cells per gonad arm (see Materials and methods) for 5 days in control (solid lines) and starvation conditions (dotted lines). The average number of germ cells per gonad arm is shown with the SEM. (E-F) The percentage of germ cell corpses among the total germ cells per gonad arm in fed animals (solid lines) and during starvation (red lines) conditions was calculated.

Germ cell apoptosis can be triggered by DNA damage or meiosis defects via CEP-1, the *C*. *elegans* p53 homologue [21–24]. *cep-1* mutant animals do not show increased germ cell apoptosis after DNA damage or meiosis defects. To test whether the germ cell apoptosis during prolonged starvation is regulated by DNA damage or meiosis defects, we quantified germ cell apoptosis in a *cep-1* mutant background [25]. We starved mid-L4 *ced-1*:*gfp* and *cep-1(gk138);ced-1*:*gfp* animals for 5 days and scored the number of germ cell corpses and the number of germ cell nuclei per gonad arm daily (see Methods). *ced-1*:*gfp* and *cep-1(gk138);ced-1*:*gfp* animals showed similar germ cell apoptosis levels in control and starvation conditions (Fig 8A), and the number of germ cell nuclei was progressively reduced during starvation (Fig 8C). Our results indicate that the proportion of germ cells eliminated by cell death was similar in the two genetic backgrounds (Fig 8E) suggesting that the increased germ cell apoptosis observed during ARD is not a consequence of DNA damage or defects during meiosis.

In our previous work, we found that *lin-35*/Rb is essential for inducing germ cell apoptosis during starvation [26]. Hence, we asked whether the high germ cell death triggered during ARD depended on *lin-35* and we found that it partially did. To demonstrate this, we subjected *ced-1*::*gfp* (control) and *lin-35(n745);ced-1*::*gfp* animals to 5 days of starvation beginning in the mid-L4 stage and determined germ cell apoptosis dynamics during ARD (Fig 8B). As previously reported [27], we found that *lin-35(n745);ced-1*::*gfp* mutant animals showed lower germ cell apoptosis compared to *ced-1*:*gfp* animals in well-fed conditions (Fig 8B). As previously reported [26], we also observed that after 24h of starvation, *lin-35(n745);ced-1*::*gfp* animals do not increase their number of germ cell corpses (Fig 8B). However, it is important to remember that during prolonged starvation the number of germ cells progressively decreased (Fig 8D), which could explain why we observed lower apoptosis levels in the *lin-35* mutant background on subsequent days (Fig 8B). We calculated the proportion of germ cells eliminated by apoptosis versus the germ cell number as we did previously (Fig 7C) and observed that germ cell apoptosis increased in the *lin-35* mutant background although it never reach control levels (Fig 8F). We concluded that *lin-35* is partially responsible for germ cell apoptosis induction during ARD, but there is an unknown mechanism that also triggers germ cell apoptosis under these conditions.

### The gonads of *daf-2* deficient animals shrink in the presence of food similar to the oogenic germline starvation response

In an attempt to reveal the molecular mechanisms that govern the oogenic germline starvation response, we subjected mid-L4 hermaphrodites of different genetic backgrounds to starvation for 5 days, followed by refeeding for 3 days, to test the capacity of their gonad to shrink and regenerate. We chose the gene candidates based on their known roles in the stress response or in germ cell proliferation. We tested the *C*. *elegans* retinoblastoma ortholog *lin-35*/Rb because it is essential for inducing apoptosis in short-term starvation [26] and partially required for inducing germ cell apoptosis in ARD (this work). We tested the *daf-16*/FoxO transcription factor due to its implication in the regulation of dauer diapause [28, 29]; the *skn-1/*NrF and *pha-4*/FoxA transcription factors due to their roles in dietary restriction and other types of stress responses [30]; the *alg-1* Argonaut ortholog due to its role in microRNAs pathway regulation and germ cell proliferation [13]; the *rsks-1* putative ribosomal protein S6 kinase, which has been implicated in resistance to starvation and, together with the eIF4E/*ife-1*, which regulates the germline cell cycle progression during development [31, 32]; the gene encoding the TIS11-like zinc-finger-containing protein *gla-3* mutant, which exhibits increased germline apoptosis [33]; and the p53 *C*. *elegans* ortholog *cep-1*, which is required for the DNA damage response [34]. None of the tested genes were required to shrink the gonad during the oogenic germline starvation response or to regenerate upon refeeding (Table 7).

**Table 7:** The oogenic germline starvation response in different genetic backgrounds.

Genotype	Relevant function for this study	References	Human ortholog	Starvation response	Re-feeding
WT	Control	[7, 8]	-	Shrunken gonad	Completely recovered
*ced-3(n1286)*	The only effector caspase	[7, 20]	Isoform 1 Caspase-2	Shrunken gonad	Completely recovered
* *				Not required	Not required
*ced-3(n717)*	The only effector caspase	[7, 20]	Isoform 1 Caspase-2	Not required	Not required
*ced-3(ok2734)*	The only effector caspase		Isoform 1 Caspase-2	Not required	Not required
*lin-35(n745)*	Causes starvation-induced germline apoptosis	[26]	Rb	Not required	Not required
*daf-16(mgDf50)*	Regulates dauer diapause	[28]	FoxO	Not required	Not required
*skn-1(zu135)*	Dietary restriction and other stresses response	[30]	Nrf	Not required	Not required
*pha-4(zu225)*	Dietary restriction and other stresses response	[30]	FoxA	Not required	Not required
*alg-1(gk214)*	miRNA pathway and proliferation	[13]	Argonaut	Not required	Not required
*rsks-1(ok1255)*	Increases resistance to starvation	[31]	Putative ribosomal protein S6K	Not required	Not required
*ife-1(bn127)*	Regulates germline progenitor number	[32]	eIF4e	Not required	Not required
*gla-3(op212)*	Increased germline apoptosis	[33]	-	Not required	Not required
*cep-1(gk138);ced-1*::*gfp*	Regulates DNA-damage-induced germline apoptosis	[34]	p53 transcription factor	Not required	Not required

Hermaphrodites from the different backgrounds were selected at the mid L4, starved for 5 days and observed daily under Nomarski microscopy to compare their gonad size to that under the oogenic germline starvation response using WT hermaphrodites as a control. At day 5, the hermaphrodites were refed for 3 days and observed daily under Nomarski microscopy to determine their gonad recovery, using WT hermaphrodites as a control.

The insulin/insulin receptor signaling (IIS) pathway has been established as a link between nutrient availability and homeostasis in species ranging from worms to humans. This pathway is maintained through the activity of insulin-like molecules that bind to the insulin receptor. In *C*. *elegans*, more than 40 peptides related to insulin bind to the insulin receptor/DAF-2 to negatively regulate the DAF-16/FoxO transcription factor. Thus, mutations in the insulin receptor/DAF-2 promote the transcription of target genes through the activity of DAF-16/FoxO to cope with nutrient scarcity and other types of stress [35]. This pathway regulates entry to dauer diapause, a well-studied diapause stage in *C*. *elegans* that occurs between larval stages L1-L2 in response to different cues, including a lack of food [36].

**Fig 9:**
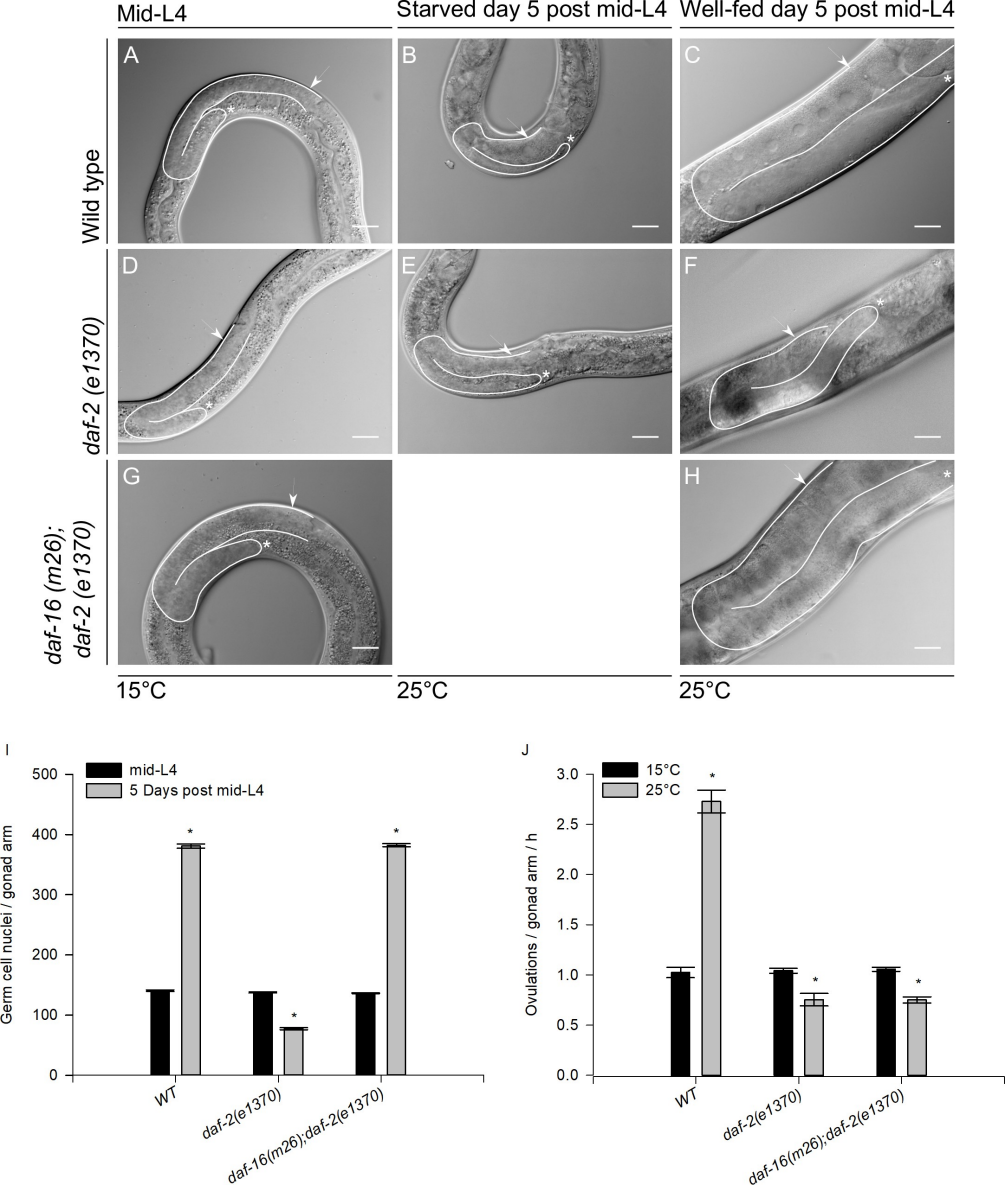
Long-term IIS pathway inactivation mimics the entry to ARD in the presence of food. Mid-L4 wild-type, *daf-2(e1370)* and *daf-16(m26); daf-2(e1370)* larvae were fed until mid-L4 at 15°C (A, D and G, respectively). Wild-type and *daf-2(e1370)* mid-L4 larvae starved for 5 days at 25°C (B, E). Nomarski images of well-fed wild-type, *daf-2(e1370)* and *daf-16(m26); daf-2(e1370)* animals grown until mid-L4 at 15°C, then upshifted to 25°C for 5 days (C, F and H). In all images one gonad arm is outlined in white; the distal gonad is marked with an asterisk (*); and the arrow points to the proximal gonad. Scale bar = 20 μm. (I) Quantification of the number of germ cell nuclei per gonad arm during prolonged IIS pathway inactivation. Animals were grown from L1 to the mid-L4 stage at the permissive temperature and then upshifted to the restrictive temperature for 5 days. Dissected gonads were stained using DAPI in each condition. The graph shows the average number of germ cell nuclei per gonad arm (±SEM). (J) Ovulation rate determination during IIS pathway inactivation. Animals were grown from L1 to the mid-L4 stage at the permissive temperature and then either upshifted to the restrictive temperature or maintained in the permissive temperature for 1 day. Animals were individually picked and scored for the number of embryos *in utero*, then placed in plates for 4 h and finally scored for the number of embryos *in utero*. The ovulation rate was calculated as follows: (final number of embryos—initial number) / (2 * 4 h). The graph shows the average number of ovulations per gonad arm per hour (±SEM).

To test the role of *daf-2* in the oogenic germline starvation response, we used the temperature-sensitive mutant *daf-2(e1370)* [37]. Synchronous populations of wild-type or *daf-2(e1370)* animals were grown from hatching to mid-L4 at the permissive temperature (15°C) (Fig 9A and 9D, respectively) and then transferred to the restrictive temperature (25°C) for 5 days in the absence of food. We found that the gonads of *daf-2(e1370)* mutant animals grown at the restrictive temperature (25°C) and starved for 5 days were able to shrink similarly to those of the wild-type animals (Fig 9E and 9B; Table 7) suggesting that *daf-2* is not required for the oogenic germline starvation response.

Unexpectedly, we observed that the well-fed control mid-L4 *daf-2* mutant animals grown at the restrictive temperature for 5 days exhibited shrunken gonads in the presence of food (Fig 9F) when their gonads were compared to those of the wild-type animals (Fig 9C). We decided to quantify the germ cell number per gonad arm at the mid-L4 stage and 5 days after IIS pathway inactivation in the presence of food using *daf-2(e1370)* mutant animals and the wild-type as a control. We found that in wild-type animals, the germ cell number increased from 140.6 ± 1.2 at mid-L4 to 380.8 ± 3.4 5 days post-mid-L4. However, *daf-2(e1370)* mutant animals did not show the same increase; instead, the number of germ cell nuclei per gonad arm was reduced from 137.6 ± 1.0 at mid-L4 to 77.2 ± 1.5 5 days post-mid-L4 (Fig 9I and Table 8).

**Table 8:** Quantification of germ cell number per gonad arm in different genetic backgrounds on food.

						* *		5 Days post mid-L4				
	mid-L4			N	15°C			N	25°C			N
WT	140.6	±	1.2	37	339.0	±	2.0	35	380.8	±	3.4	20
*daf-2(e1370)*	137.6	±	1.0	42	337.0	±	1.4	44	77.2	±	1.5	41
*daf-16(m26);daf-2(e1370)*	136.4	±	0.7	43	342.3	±	1.7	43	382.2	±	2.5	22

The gonads of animals were dissected, stained and scored for the number of germ cells using epifluorescence microscopy. Animals were grown in NGM plates seeded with OP50 from hatching to mid-L4 at 15°C and then incubated on food at 15°C or 25°C for 5 days. Animals were picked and dissected. The dissected gonads were stained with DAPI and the number of germ cells per gonad arm was scored under fluorescence microscopy.

The transcription factor *daf-16/FoxO* is activated when the *daf-2* signal is absent [38]. As we previously showed, *daf-16* is not required to shrink the gonad during ARD, but we tested whether gonad shrinking induced in the absence of *daf-2* in the presence of food requires *daf-16*. To test this, *daf-16(m26);daf-2(e1370)* double mutant animals were grown from hatching to mid-L4 on food at the permissive temperature and then shifted to 25°C for 5 days (Fig 9G and 9H). We observed that the gonads of animals lacking *daf-2* did not shrink in the presence of food when *daf-16* was not present. We quantified the germ cell number in this double mutant genetic background and found that the number of germ cells increased from 136.4 ± 0.7 at mid-L4 to 382.2 ± 2.5 5 days after IIS pathway inactivation in the presence of food (Fig 9I and Table 8). These results suggest that inactivation of *daf-2* in well-fed animals might trigger a mechanism similar to ARD that requires *daf-16*/FoxO.

To test whether IIS pathway inactivation could mimic entry to ARD even in the presence of food, we determined the ovulation rate in 1-day-old wild-type and *daf-2(e1370)* and *daf-16(m26);daf-2(e1370)* mutant animals grown from L1 to mid-L4 at 15°C that were late upshifted to 25°C in the presence of food. It has been reported that ARD causes a delay in the ovulation rate [8]. Wild-type animals ovulate more frequently when upshifted to 25°C (N = 30) (Fig 9J). In contrast, *daf-2(e1370)* animals showed no increase in the ovulation rate when upshifted to 25°C (N = 33). However, *daf-16(m26);daf-2(e1370)* mutant animals continued to exhibit a slowed ovulation rate (N = 33) (Fig 9J).

Another feature of the oogenic germline starvation response is that the ovulation rate is limited by the rate of oocyte growth [8]. Starved animals almost always form only a single oocyte at a time in each gonad arm ([8], Figs 1F and 9B). However, gonads from *daf-2(e1370)* animals grown at 25°C in the presence of food did not exhibit this phenotype (Fig 9E). We concluded that DAF-2 might participate in the oogenic germline response but that it is not the only signal that participates in this phenomenon. It is also possible that because the *daf-2* mutation results in pleiotropic phenotypes, it is not easy to study its role in the oogenic germline response.

## Discussion

Angelo and van Gilst (2009) reported a novel type of adult reproductive diapause for the first time while studying reproduction when nutrients are limited. They showed that crowded populations of mid-L4 larval stage animals subjected to starvation delay their reproductive cycle and are able to live for up to 30 days without food. Furthermore, when conditions are restored, these animals resume their reproductive cycle and live out a normal lifespan. During ARD, the gonad experiences reversible germ cell loss and size reduction; when animals are returned to food, the gonad regenerates in terms of both germ cell number and size [7].

Later, Seidel and Kimble (2011) studied ARD in depth. They showed that germline shrinkage occurs in all oogenic germlines, from the mid-L4 to adult stages, and practically all shrunken gonads regenerate upon refeeding if bagging is prevented. Seidel and Kimble (2011) showed that embryo production continues during prolonged starvation, and it is possible to distinguish one or two viable-looking embryos within the uterus, which are a product of recent fertilizations, indicating that oocyte production and fertilization are active but considerably delayed in starved animals; however, embryo viability is severely impaired during starvation. They also showed that population density is not a prerequisite for inducing or maintaining the starvation response in starved animals and suggested that this phenomenon is not a form of diapause [8].

Despite not supporting the existence of the ARD proposed by Angelo and van Gilst (2009), Seidel and Kimble (2011) coined the term “oogenic germline starvation response” referring to the germline plasticity controlled by nutritional cues [8]. They also reported that starvation causes reversible cell-cycle quiescence in adult germline stem cells, reducing the number of M-phase cells under 30 minutes of starvation and rapidly re-establishing their number upon refeeding [9].

Our results support the following conclusions: Long-term exposure to fasting compromises the animals’ reproductive capacity, and the oogenic germline starvation response prevents oogenic germ cells from undergoing prolonged arrest in diakinesis by slowing oocyte production. In contrast to Angelo and van Gilst (2009), we demonstrated that apoptosis is not the cause of gonad shrinkage during ARD and that it is not essential for fertility recovery after ARD. Instead, we propose that once germ cell proliferation stops, ovulation contributes to gonad shrinking during the oogenic germline starvation response. Germ cell apoptosis is increased during ARD and continues during prolonged starvation. Germ cell apoptosis during ARD is not induced by DNA damage or chromosome segregation errors during meiosis but is partially regulated by *lin-35*/Rb. We also found that during ARD an unknown mechanism triggers germ cell apoptosis. Finally, we suggest that inactivation of the IIS signaling pathway triggers gonad shrinking in the presence of food that might resemble the oogenic germline response.

### ARD compromises fertility by affecting oogenic germ cells

In the present work, we showed that fertility is affected after ARD (Figs 2, 3 and 6). Although recovered animals exhibit restoration of fertility upon refeeding, none of the genetic backgrounds tested (N2, *fog-1*, *fog-2* and *ced-3*) produced broods that were as large as those of the controls by self-fertilization or mating. Angelo and van Gilst (2009) proposed that the fecundity of self-fertilizing animals depended on the survival of functional sperm during starvation [7]. However, we observed that, at least during the first 5 days of starvation, the sperm number remained unchanged.

Several authors have used embryonic lethality to reflect oocyte quality [3, 39]. Our results showed that animals produce more dead embryos when they use their self-sperm to fertilize oocytes after recovery from 5 days of starvation than their respective controls (Figs 2 and 6). We showed that recovered self-fertilizing hermaphrodites produce dead embryos during the entire reproductive period (Fig 2). However, recovered *fog-1(q253)* and *fog-2(q71)* females produced the majority of dead embryos during the first two days after mating (Fig 3), which suggests that the self-sperm exposed to starvation and used to fertilize oocytes in wild-type animals had been compromised. Moreover, when well-fed male sperm were provided, the oogenic germ cells produced during starvation were prone to produce dead embryos after fertilization. Our results suggest that the self-fertilizing capacity of hermaphrodites is not only compromised by the sperm number, but also by oogenic germ cell exhaustion after ARD. It is worth noting that the effects of ARD on sperm capacity remain unclear. Further studies are needed to determine the effect of ARD on sperm quality.

### Oogenesis arrest is prevented by the oogenic germline starvation response

In the *C*. *elegans* germline, the presence of food and sperm promotes meiotic progression and oogenesis. Sperm depletion causes prolonged diakinesis arrest in the proximal gonad due to oocyte stacking, and starvation halts meiotic progression in the pachytene region [40]. In replete conditions and the presence of sperm, hermaphrodites ovulate every ~20 minutes per gonad arm, while during ARD, hermaphrodites exhibit slowed oogenesis and produce one oocyte at a time, and the next oocyte production starts once the fully grown oocyte is ovulated, which occurs approx. every ~8 h per gonad arm, regardless of the presence of sperm [8]. The oocytes that experience longer arrest in diakinesis are more likely to produce dead embryos [3]. We demonstrated that at day 5 post-mid-L4, in the presence of food, *fog-1* and *fog-2* animals actively produced and stacked as many oocytes as possible, while under starvation conditions *fog-1* and *fog-2* animals produced and stacked fewer oocytes within their gonads (Fig 4). When mated, well-fed 6-day-old *fog-1* and *fog-2* animals produced smaller broods and more dead embryos than *fog-1* and *fog-2* animals recovered from starvation, suggesting that slowing oogenesis during starvation is a beneficial mechanism for preserving the reproductive capacity and oocyte quality.

### Apoptosis is important to preserve oocyte quality during ARD; however, it is not essential for fertility and gonad shrinking

In this study, we demonstrated that *ced-3* mutant animals respond to starvation by shrinking their gonads during ARD and partially recover their fertility upon refeeding (Figs 5 and 6). In contrast to Angelo and van Gilst (2009), who reported that apoptosis-deficient *ced-3(n1286)* animals subjected to 15 days of starvation did not show loss of germ cell nuclei or gonad shrinkage, and did not produce progeny after ARD [7]. In fact, we observed that wild-type animals and *ced-3* deficient mutants are unable to produce progeny after spending 15 days under starvation, either by self-fertilization or mating with well-fed wild-type males.

As previously reported [3], we observed that apoptosis-deficient mutants produced fewer offspring and exhibited higher embryonic lethality under control conditions compared to the wild-type animals. In this paper, we show that the apoptosis-deficient animals that were recovered from 5 days of starvation produced even fewer offspring and exhibited higher embryonic lethality than the wild-type animals. Our results suggest that apoptosis is important to preserve oocyte quality in control or prolonged starvation conditions.

### Germ cell apoptosis continues during ARD

Our work shows that apoptosis is increased during prolonged starvation and continues to be high during ARD. Germ cell apoptosis is required during oogenesis for properly allocating resources within the gonad and ensuring oocyte quality [3, 41]. Apoptosis increases with age under control conditions and continues in later life contributing to gonad degeneration [41, 42]. We found that increased germ cell apoptosis during ARD is not triggered by *cep-1*/p53, suggesting that it is not caused by DNA damage or meiotic defects.

Furthermore, germ cell apoptosis can be triggered when animals face different types of stress [19]. Previously, we reported that one-day-old animals exposed to 6 h of starvation presented a 2-fold increase in the number of germ cell corpses, for which *lin-35*/Rb is responsible [19, 26]. Unexpectedly, we found that apoptosis seems to be induced during ARD by *lin-35*/Rb and an unknown mechanism.

Why is germ cell apoptosis elevated during ARD? Our results support the notion proposed by Andux and Ellis (2008) that germ cell death serves as a mechanism to redistribute resources in the gonad [3]. The biological significance of this mechanism might be that it ensures oocyte production only under suitable conditions. At the mid-L4 stage when ARD entry is promoted, some germ cells have already committed to the meiotic oogenic cell fate, which is irreversible; however, completion of meiosis and development into a mature oocyte takes several hours. Thus, we think that the germ cell apoptosis observed during ARD might arise from the germ cells that escape the mitotic cell fate but, due to lack of resources, are eliminated by apoptosis.

### DAF-2 inactivation causes gonad size reduction in the presence of food

We sought to investigate the molecular mechanism that governs the oogenic germline starvation response during ARD. We tested some genes involved in the regulation of dauer diapause, L1 arrest and other genes implicated in germ cell apoptosis and proliferation and demonstrated that only IIS in the mid-L4 larval stage caused a reduction in gonad size even in the presence of food (Table 8 and Fig 9). We propose that IIS pathway inactivation might trigger a response that mimics the entry into ARD in well-fed conditions, since animals show a reduced gonad size (Fig 9). Similarly, *daf-2* animals slow ovulation when they reduced their gonad size suggesting that *daf-2* might participate in ARD signaling; however, the shrunken gonads of *daf-2* animals do not form one single oocyte at a time as that of the ARD gonads, and *daf-2* is not essential for the gonad shrinking during ARD. Although our results provide some evidence of the pathway that governs the oogenic germline starvation response, further information is still needed for a better understanding of the precise molecular mechanism that controls reproduction under starvation conditions.
